# Spindle Cell Carcinoma Presenting as a Massive Pleural Effusion

**DOI:** 10.7759/cureus.54526

**Published:** 2024-02-20

**Authors:** Moutaz Ghrewati, Anas Mahmoud, Malina Mohtadi, Joseph Russo, Mohammad Alnabulsi, Mourad Ismail, Mehandar Kumar

**Affiliations:** 1 Oncology, St. Joseph’s University Medical Center, Paterson, USA; 2 Internal Medicine, St. Joseph’s University Medical Center, Paterson, USA; 3 Pulmonary and Critical Care, St. Joseph’s University Medical Center, Paterson, USA

**Keywords:** chemotherapy response, non small cell lung cancer, pulmonary sarcomatoid carcinoma (psc), lung cancer, spindle cell sarcoma

## Abstract

The lung's sarcomatoid carcinomas (SC) are a heterogeneous sporadic group of non-small cell lung carcinomas (NSCLCs) and are very challenging to diagnose and treat. Spindle cell carcinoma (SpCC) is a very rare subset of this group. Hence, the prognosis and treatments are unclear due to the limited literature available. The presentation of this cancer varies based on the site of the neoplasm and the complications and metastases observed at the time of diagnosis. Here, we report a 73-year-old man who presented to the emergency room after two months of worsening dyspnea and fatigue. Chest X-ray showed an extensive left-sided pleural effusion. A computed tomography (CT) scan of the chest showed a pleural-based mass that came back as SpCC, for which he was referred to a university hospital.

## Introduction

The lung's sarcomatoid carcinoma (SC) is an uncommon form of non-small cell carcinoma (NSCLC) characterized by its rarity, high-grade nature, and poor differentiation [1]. SC constitutes a diverse group of tumors that lack clearly defined diagnostic criteria [2]. The World Health Organization (WHO) classifies SC into five main types based on histological features: pleomorphic carcinoma, spindle carcinoma, giant cell carcinoma, carcinosarcoma, and pulmonary blastoma [3]. Spindle cell carcinoma (SpCC) is characterized as an infrequent histological subtype of SC, composed of tumor cells with a spindle-shaped morphology [4]. A retrospective study of 718 cases found that SpCC represented only 0.4% of all lung malignancies [5]. Unfortunately, limited information is available regarding the clinical presentation, progression of the disease, and treatment for SpCC. Here, we attempt to shed light on another case of SpCC in an elderly male. 

## Case presentation

Our patient is a 73-year-old male with no past medical history who presented to the emergency room (ER) complaining of progressively worsening shortness of breath (SOB) and fatigue for two months duration. He reported an increase in heartburn with food regurgitation that had started two months earlier. Over-the-counter (OTC) bismuth subsalicylate was taken, but it did not provide relief. The fatigue had worsened to the extent that he was unable to climb a flight of stairs without experiencing dyspnea. Even simple activities, such as walking around at home, became challenging, prompting him to seek assistance at the emergency room. The patient immigrated from Peru 40 years ago and did not leave the United States since then. He denied any chest/back/abdominal pain, recent infection, cough, fever, weight loss, or difficulty swallowing. The patient had smoked in his twenties only for five years, with four to five cigarettes per day. He also used to drink occasionally, but his last drink was more than 12 years ago. The patient reported living with 10 cats in his apartment, and he used to work in a junkyard where he dismantled cars.

**Figure 1:**
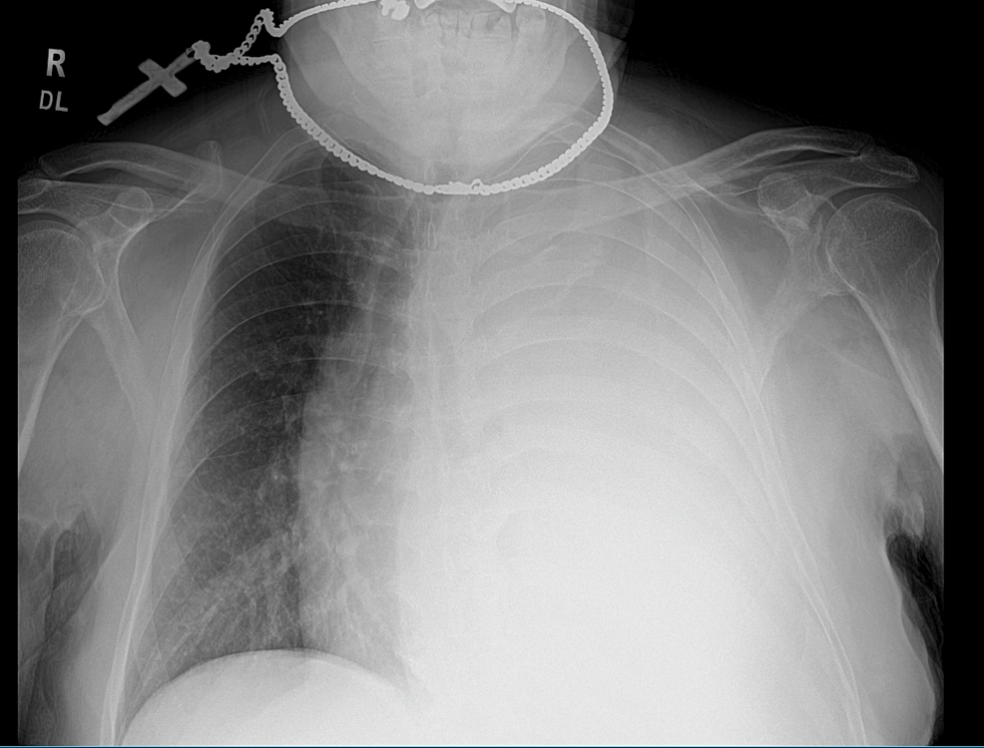
Chest X-ray showing complete opacification of the left lung most likely due to left-side pleural effusion.

**Figure 2:**
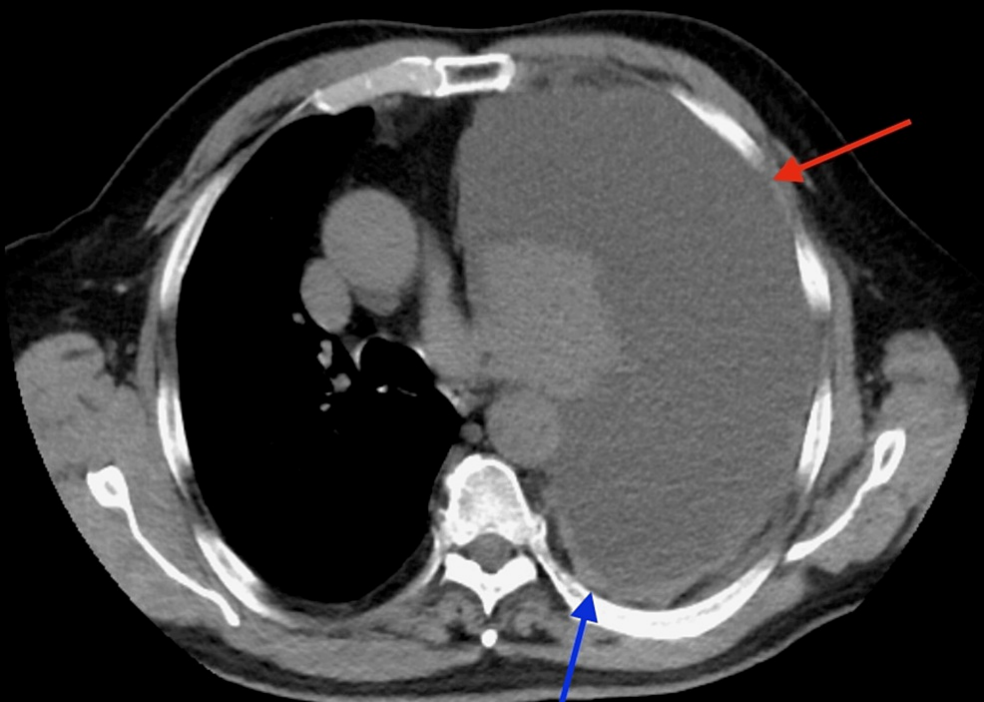
CT chest without contrast showing a large left-side pleural effusion (red arrow) with possible underlying pleural-based masses (blue arrow).

**Figure 3:**
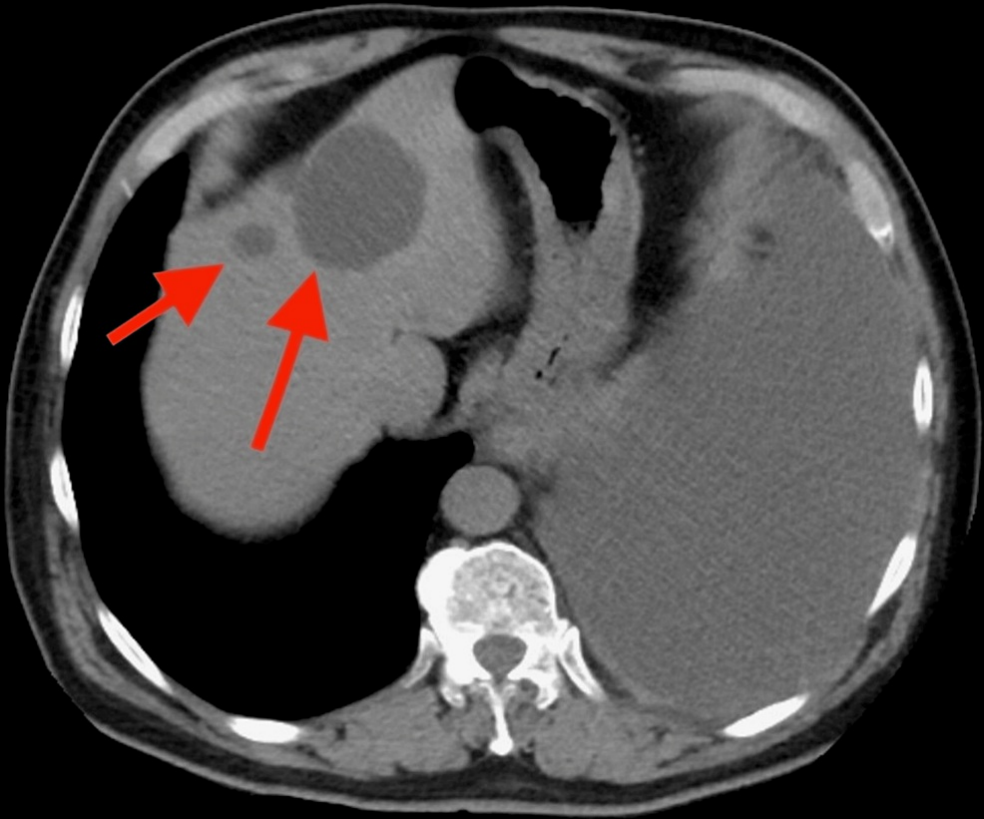
CT chest without contrast showing the upper part of the abdomen. CT without contrast shows multiple liver masses most likely neoplasms (red arrow).

**Figure 4:**
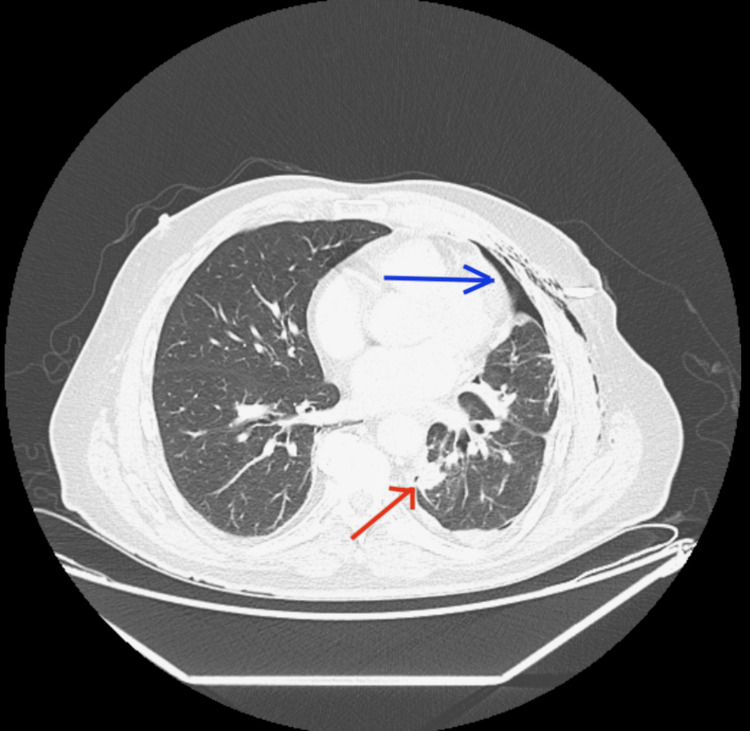
CT chest with contrast showing pleural-based masses in the left lung (red arrow) and small pneumothorax (blue arrow).

In the ER, his vitals were stable. A chest X-ray showed a complete collapse of the left lung secondary to a large left-side pleural effusion (Figure 1). A CT scan of the chest without contrast showed the collapse of the left lung secondary to extensive pleural effusion, a left hilar mass causing narrowing of the airways, and a 1.8 cm pleural-based mass on the left (Figure 2). It also showed lesions in the liver (Figure 3). Immediately, a pigtail thoracotomy tube drained 3 L of serosanguineous fluid, and the patient reported immediate significant relief. The fluid analysis showed a lymphocytic predominance of white blood cells. A CT scan of the chest and abdomen with contrast revealed multiple mass-like densities in the left lung base, suggesting a neoplasm. In addition, a 2.6 cm x 2 cm pleural-based density in the left lower lobe, possibly indicative of a neoplasm, was identified. A small pneumothorax was also observed that resolved within two days following chest tube insertion (Figure 4).

Interventional radiology (IR) guided biopsy of the mass was conducted, and a tissue sample was sent to the pathology. The pathology came back showing a spindle cell neoplasm. Immunostaining was negative for pan-cytokeratin, EMA, p40, S100, BCL2, CD117, beta-catenin, and MUC4 and only positive for vimentin. Immunostaining for transducin-like enhancer of split 1 (TLE-1) was conducted to rule out synovial sarcoma, and the results were positive. However, subsequent testing using fluorescence in situ hybridization (FISH) analysis for SS18 (SYT) (18Q11.2) returned negative results, leading to the exclusion of synovial sarcoma. The oncology team supported and recommended transferring the patient to a university hospital due to the rarity of this type of cancer.

## Discussion

Upon a literature review, very few cases of SpCC have been reported, and most are discovered incidentally. This was also the scenario with our patient, who presented with increasing shortness of breath and fatigue. Typical presentations can include chest pain, cough, shortness of breath, or hemoptysis [1]. SpCC typically presents in elderly males, with a strong association with a history of smoking [6,1]. Most SpCCs are identified in the periphery of the upper lobe [1,7]. 

As aforementioned, SC is a type of NSCLC that is poorly differentiated [1]. SpCC consists of almost an entire population of epithelial spindle cells with no other carcinomatous characteristics [4]. However, the proper diagnostic challenge is further differentiating SC from malignant mesothelioma, as they can share a similar presentation of morphology [3]. Immunohistochemistry is required to differentiate between the two; keratin positivity confirms the sarcomatoid nature of a malignancy rather than one of mesothelioma or malignant melanoma [3].

Sarcomas can also be primarily in the lung or metastatic to the lung or pleura. Thus, it is also imperative to exclude the possibility of synovial sarcoma [3]. Synovial sarcoma shows positive immunohistochemical staining for TLE-1 and is characterized by the distinct cytogenetic translocation of t(X; 18)(p11; q11) involving SSX genes [8,9]. Therefore, it is essential to note that in our case, staining for TLE-1 was performed to rule out synovial sarcoma and was positive. However, additional testing using FISH analysis of SS18 (SYT) (18Q11.2) yielded a negative result, thereby ruling out synovial sarcoma [9]. In SpCC, the cells can appear morphologically with a wide breadth of variety, from epithelioid to only spindles or disorganized fascicles [3]. Immunohistochemistry evaluation of these malignancies will appear positive for cytokeratin and vimentin and negative for desmin, S-100, α-smooth muscle actin, and CD34 [10]. 

The five-year survival rate for all sarcomatoid lung cancers is about 20% [8]. Interestingly, studies show advanced SpCC has minimal sensitivity to chemotherapy in overall survival compared to no treatment [6]. Reports have found that chemotherapy and radiation are not sufficient in inoperable cases; therefore, surgical resection is preferred [8]. This further solidifies the importance of early diagnosis of such malignancies, which can be monitored in guideline-appropriate low-dose chest CT screenings.

## Conclusions

Here, we present a case of SpCC in an elderly male with NSCLC that turned out to be SpCC. SpCC accounts for only a very minimal percentage of all lung cancers and is often difficult to diagnose without appropriate histology and immunochemistry analysis to distinguish it from other malignancies correctly. Presentation is similar to all lung cancers where chest pain, cough, shortness of breath, or hemoptysis can happen. The prognosis of all SCs is very dismal as they have minimal sensitivity to chemotherapy. Thus, early diagnosis and surgical resection are paramount. We hope this case provides insights into the presentation and appropriate diagnostic workup in identifying this rare malignancy appropriately. 
